# Calponin-Like Chd64 Is Partly Disordered

**DOI:** 10.1371/journal.pone.0096809

**Published:** 2014-05-07

**Authors:** Małgorzata Kozłowska, Aneta Tarczewska, Michał Jakób, Kamil Szpotkowski, Magdalena Wojtas, Grzegorz Rymarczyk, Andrzej Ożyhar

**Affiliations:** 1 Department of Biochemistry, Faculty of Chemistry, Wrocław University of Technology, Wrocław, Poland; 2 Center for Biocrystallographic Research, Institute of Bioorganic Chemistry, Polish Academy of Sciences, Poznań, Poland; Hungarian Academy of Sciences, Hungary

## Abstract

20-hydroxyecdysone (20E) and juvenile hormone (JH) signaling pathways interact to regulate insect development. Recently, two proteins, a calponin-like Chd64 and immunophilin FKBP39 have been found to play a pivotal role in the cross-talk between 20E and JH, although the molecular basis of interaction remains unknown. The aim of this work was to identify the structural features that would provide understanding of the role of Chd64 in multiple and dynamic complex that cross-links the signaling pathways. Here, we demonstrate the results of *in silico* and *in vitro* analyses of the structural organization of Chd64 from *Drosophila melanogaster* and its homologue from *Tribolium castaneum*. Computational analysis predicted the existence of disordered regions on the termini of both proteins, while the central region appeared to be globular, probably corresponding to the calponin homology (CH) domain. *In vitro* analyses of the hydrodynamic properties of the proteins from analytical size-exclusion chromatography and analytical ultracentrifugation revealed that DmChd64 and TcChd64 had an asymmetrical, elongated shape, which was further confirmed by small angle X-ray scattering (SAXS). The Kratky plot indicated disorderness in both Chd64 proteins, which could possibly be on the protein termini and which would give rise to specific hydrodynamic properties. Disordered tails are often involved in diverse interactions. Therefore, it is highly possible that there are intrinsically disordered regions (IDRs) on both termini of the Chd64 proteins that serve as platforms for multiple interaction with various partners and constitute the foundation for their regulatory function.

## Introduction

Two major lipophilic hormones regulate insect development and growth, a steroid called 20-hydroxyecdysone (20E) and the sesquiterpenoid juvenile hormone (JH). The initiation of molting and other essential biological processes are controlled by 20E, whereas JH works as a modulator of the ecdysteroid-induced gene expression cascade [1], [2]. In contrast to 20E, whose mode of action is well-known, the biological mechanism of gene regulation by JH is poorly understood. Consequently, the cross-talk between the two signaling pathways remains a puzzle. Recently, Li *et al.* [3] identified a common 29-nucleotide JH-response element (JHRE) in the promoter regions of 13 out of 16 genes regulated by JH in *Drosophila melanogaster* L57 and *Apis mellifera* brain cells. Li *et al.* [3] also identified two nuclear proteins, 21 kDa calponin-like protein (Chd64) and 39 kDa FK506-binding protein (FKBP39). It has been shown that these two proteins bind to JHRE as well as some other nuclear proteins including: ecdysone receptor (EcR), ultraspiracle (Usp) and methoprene-tolerant (Met). Suppression of the induction of Chd64 and FKBP39 by specific dsRNA prevented JH induction of the JHRE-dependent reporter, suggesting that these proteins are necessary for JH action. Based on their findings, the researchers proposed a model in which Chd64 and FKBP39 are part of a multi-protein complex that mediates the cross-talk between JH and 20E [3]. In another study, Liu *et al.* [4] reported that the Chd64-related protein HaCal from *Helicoverpa armigera* has also been found to participate in the cross-talk between hormonal signaling pathways from 20E and JH [4].

Chd64 belongs to the family of proteins containing a calponin homology (CH) domain. The CH domain is a proteinous module found throughout many species. It is defined by a number of invariant core residues, which may indicate evolutional conservation [5], [6]. Although the conformation of the primary chain of the domain is similar for most family members, proteins that contain the CH domain differ in the number of CH repeats and the presence of additional defined sequences, e.g. the EF hand, calponin-family repeat (CFR), or actin binding domain (ABD). Two tandem CH domains constitute an actin-binding region in proteins involved in F-actin cross-linking, e.g. spectrin, α-actinin, dystrophin and fimbrin [7], [8]. As a single copy, the CH domain is found in proteins that participate in muscle contraction such as calponin and transgelin (also known as SM22) and in signal-transduction proteins such as Vav and IQGAPs (GTPase-activating proteins that contain calmodulin-binding IQ motifs) [5]. DroVav, a protein in *Drosophila* that contains the CH domain, was suggested to play a critical role as a signal transducer during fruit fly development [9]; nevertheless, the exact role of the CH domain is not well understood [5], [6], [10].

Until now, Chd64 has not been biochemically or structurally characterised. Our goal was to identify the structural features that would provide understanding of the role of Chd64 in the multiple and dynamic protein complex. Proteins that form and participate in multi-protein complexes frequently participate in highly specific interactions with multiple, structurally diverse partners. In recent years, it has been shown that this feature is highly connected with intrinsically disordered proteins (IDPs) [11]. IDPs almost completely lack a well-defined tertiary structure under physiological conditions and are characterised by extraordinary structural flexibility and plasticity [12]. These features are believed to represent a major functional advantage for this group of proteins, enabling them to form large surfaces for interaction with structurally diverse partners. IDPs or intrinsically disordered regions (IDRs) which also exist in largely structured proteins are most likely involved in the regulatory functions of their binding partners and promote the assembly of supramolecular complexes [13]. According to the model proposed by Li *et al.* [3] Chd64 participates in the formation of multi-protein complex and through interaction with various partners mediates the cross-talk between the hormonal signaling pathways.

We decided to conduct comparative, parallel biochemical studies on Chd64 from *Drosophila melanogaster* and its homologue from the non-related organism *Tribolium castaneum*. The red flour beetle, *T. castaneum,* exhibits a classic developmental response to JH. Feeding its larvae by JH agonists results in prolonged larval development leading to the production of supernumerary “giant” larvae which fail to pupate [14], [15]. Besides, *Drosophila* has particularly adapted to a short life cycle thus findings on *Drosophila* are not always applicable to other arthropods [16], therefore our studies have been extended to a more generic arthropod.

This paper presents the results of the structural analysis of DmChd64 and TcChd64 chain conformation performed with a range of *in silico* and *in vitro* methods. Our results indicate that the proteins may exhibit dual structural organisation, i.e. they appear to have a globular core, which probably corresponds to the CH domain and disordered termini. The presence of a disordered N- and C-termini in Chd64 may establish the conditions for its biological function since the labile, dynamic and highly adaptable nature of disordered regions enables interaction with various partners.

## Materials and Methods

### Buffers used for Purification of DmChd64 and TcChd64

All buffers were prepared at 24°C. The lysis buffer was 20 mM Na_2_HPO_4_, 150 mM NaCl, 1 mM β-mercaptoethanol, 0.2 mg/ml PMSF, pH 7.0. Buffer A was 50 mM Na_2_HPO_4_, 600 mM NaCl, 5% (v/v) glycerol, 1 mM β-mercaptoethanol, pH 7.0. Buffer B was 50 mM Na_2_HPO_4_, 600 mM NaCl, 5% glycerol, 1 mM β-mercaptoethanol, 200 mM imidazole, pH 7.0. Buffer C was 100 mM Tris, 150 mM NaCl, pH7.8. Buffer D was 100 mM Tris, 150 mM NaCl, 2.5 mM desthiobiotin, pH7.8. Buffer E was 50 mM Na_2_HPO_4_, 300 mM NaCl, 1 mM β-mercaptoethanol, pH 7.0. Buffer F was 50 mM Na_2_HPO_4_, 300 mM NaCl, 1 mM β-mercaptoethanol, 200 mM imidazole, pH 7.0. Buffer G was 50 mM Na_2_HPO_4_, 150 mM NaCl, pH 7.0.

### Construction of Expression Vectors

cDNA clones encoding the full-length DmChd64 (GH28730) and TcChd64 (NCBI Reference Sequence: XP_975100.1) obtained from Drosophila Genomics Resource Center (DGRC) and GeneArt Life Technologies, respectively, were used as templates for PCR. The *Escherichia coli* strain XL1-Blue was used for all cloning procedures (Novagen). To amplify by PCR the full-length DmChd64 and TcChd64 cDNA synthetic oligonucleotides as primers were used. The following forward and reverse primers were used for PCR amplification: 5′-gcgcgg*ggatcc*GCGTCCAACCGTGCAGCG-3′ (forward), 5′-gggcc*aagcttCATGTGCCGGGTGTTGCC*-3′ (reverse) for DmChd64 and 5′-ggcgac*ggatcc*GCAACCAATCGTGCAACC-3′ (forward), 5′-ggccgg*aagctt*CATATGACGGGTATTGCCAAAATTAATACC-3′ (reverse) for TcChd64. The forward primer sequences introduce the restriction site for *Bam*HI and the reverse primer sequences introduce the restriction site for *Hind*III. The uppercase letters in the primer sequences represent the sequence derived from DmChd64 and TcChd64 genes, whereas the lowercase letters represent nucleotides added for cloning purposes; the restriction sites are shown in italics. The obtained DNA fragments were double-digested with *Bam*HI and *Hind*III and ligated into corresponding sites of modified pQE80L(Qiagen) vectors: pQE80L-XH, pQE80L-SX and pQE80L-SXH adding the 8×His-tag on the C-terminus (XH), the Strep II-tag to the N-terminus (SX) and both of them (SXH) to the target proteins, respectively. The final expression products of DmChd64 and TcChd64 had the following amino acids added to each terminus of the coding sequences: MGS (added to the N-terminus), KLHHHHHHHH (added to the C-terminus) for pQE80L-XH contructs; MASWSHPQFEKGAGS (added to the N-terminus), KL (added to the C-terminus) for pQE80L-SX contructs; MASWSHPQFEKGAGS (added to the N-terminus), KLHHHHHHHH (added to the C-terminus) for pQE80L-SXH constructs. The presence of the inserts within the pQE80L-XH/SX/SXH vectors was confirmed by restriction analysis (data not shown) and the purified constructs were verified with DNA sequencing.

### Expression and Purification of DmChd64 and TcChd64

Prior to elaboration of the efficient purification procedure, the expression of each recombinant DmChd64 and TcChd64 variant was evaluated (data not shown). Both proteins had the same expression conditions and the first step of purification was based on the8×His-tag at the C-end of both proteins. 100 ml of the TB medium containing appropriate antibiotics: 35 µg/ml chloramphenicol and 50 µg/ml carbenicilin was inoculated with DmChd64 (containing the 8×His-tag on the C-terminus and the Strep II-tag on the N-terminus) or TcChd64 (containing the 8×His-tag on the C-terminus) transformed *Escherichia coli* strain BL21(DE3)pLysS (Novagen). After overnight incubation at 29°C and 182 rpm, the starter culture was used to inoculate 4 l of the TB medium with antibiotics (35 µg/ml chloramphenicol and 50 µg/ml carbenicilin). The final suspension was divided into 250 ml portions and incubated at 29°C and 182 rpm. When the OD_600_ of the growing culture reached a value of 0.6–0.8, synthesis of the recombinant proteins was induced by the addition of 0.25 mM of isopropyl-β-D-thiogalactopyranoside (IPTG). After 3 h of incubation, bacterial cells were collected after 10 min centrifugation at 4000×g, 4°C. Each obtained pellet was washed once with 12 ml of the lysis buffer per 500 ml of original culture, resuspended in 12 ml of the lysis buffer and frozen at −80°C. The frozen cells from 1 l of culture were lysed by thawing in a 25°C water bath and freezing twice. As soon as the second thawing had begun, the cells were supplemented with an appropriate volume of PMSF and β-mercaptoethanol to a final concentration of 0.2 mg/ml and 1 mM, respectively. Then, DNase I and RNase A were added to the final concentration of 10 µg/ml of each enzyme and the lysates were incubated on ice until there was a loss of viscosity, i.e. nucleic acids were completely digested. The cell extract was clarified by 1 hour of centrifugation at 17 500×g at 4°C. The soluble fraction was collected into Falcon 50 ml tubes and supplemented with 0.2 mg/ml PMSF, and then purified using immobilised metal ion affinity chromatography (IMAC). For both proteins, DmChd64 and TcChd64, the procedure of the first step of purification was the same; the only differences were in the buffers used (see Buffers used for purification of DmChd64 and TcChd64). The cell lysate was incubated for 1 h at 4°C, 40 rpm with 1200 µl of Co^2+^-TALON resin (Clontech), which had been previously equilibrated with buffers A for DmChd64 and E for TcChd64. The resin was then loaded on a disposable column and washed 5 times with 5 ml of buffers A or E. Subsequently, the resin was transferred into a Tricorn 5/50 column (Amersham Biosciences) and then connected to the ÄKTAexplorer (Amersham Biosciences) system and operated at 0.5 ml/min at room temperature. The column was washed with at least 5 ml of buffers A or E and then with at least 5 ml of a 10% gradient of elution buffer B for DmChd64 and F for TcChd64. The fusion protein was eluted with 5 ml of appropriate buffers, collected in 0.5 ml fractions and combined. For DmChd64, the second step of purification was affinity chromatography based on the Strep II-tag on the N-terminus of the protein. The solution containing DmChd64 obtained from the first step of purification was concentrated to a volume of 0.5 ml using an Amicon Ultra-4 Centrifugal Filter Unit (Millipore) and applied to the Strep-Trap HP (Amersham Biosciences) column equilibrated with buffer C. The column was run on the ÄKTAexplorer (Amersham Biosciences) system, and operated at 0.5 ml/min at room temperature. The column was first washed with 5 ml of buffer C. The fusion protein was eluted with 3 ml of buffer D, collected in 0.5 ml fractions and combined. Afterwards, affinity chromatography gel filtration was used. Eluted DmChd64 or TcChd64 was concentrated to a volume of 0.5 ml using an Amicon Ultra-4 Centrifugal Filter Unit (Millipore) and injected in to a single Superdex 200 10/300 GL equilibrated with buffer G (Amersham Biosciences) and two tandem Superdex 75 10/300 GL (Amersham Biosciences) columns, respectively. The columns were run on the ÄKTAexplorer (Amersham Biosciences) system and operated at 0.5 ml/min at room temperature. Fractions containing pure recombinant proteins were collected into 1.5 ml tubes, combined, aliquoted and stored at −80°C. The concentration of purified proteins was determined spectrophotometrically at 280 nm. The absorption coefficients for DmChd64 and TcChd64, calculated according to the method proposed by Gil and von Hippel [17], were 0.841 and 0.699 ml/(mg×cm), respectively. Protein content and purity was estimated after every stage of purification with SDS-PAGE and Coomasie Brilliant Blue R-250 stained gels. Electrophoretic mobility was determined for the final samples of protein preparations (see below).

### SDS-PAGE

Protein samples were analysed by SDS-PAGE using 12% polyacrylamide gels developed in a Tris/glycine system [18]. The Unstained Protein Molecular Marker (Fermentas) was used. After electrophoresis the proteins were stained with Coomassie Brilliant Blue R-250 [19]. Densitometric analysis and electrophoteric mobility of the distained gels was performed using the AIDA Bio-package software (Raytest Isotopenmeβgeräte GmbH).

### ESI Mass Spectrometry

Solutions of purified DmChd64 and TcChd64 protein were dialysed against 1% acetic acid and concentrated to approximately 1 mg/ml using the Amicon Ultra-4 Centrifugal Filter Unit (Millipore). High-resolution mass spectra were obtained on the micrOTOF-G™ spectrometer (Bruker Daltonick), equipped with an Apollo II electrospray ionization (ESI) source with an ion funnel. The protein solution was infused at a flow rate of 3 µl/min. The mass spectrometer was operated in the positive ion mode. The mass resolution was 15000 FWHM. The instrument parameters were as follows: a scan range of m/z 300–2300; dry gas: nitrogen; at a temperature of 180°C. Before performing each measurement the instrument was calibrated externally with the Tunemix mixture (Bruker Daltonik) in the quadratic regression mode. The acquisition of data was performed using DataAnalysis software from Daltonik GmbH (Bremen). The theoretical molecular mass values were calculated using ProtParam tool available at http://us.expasy.org/tools/protparam.html.

### Circular Dichroism Spectroscopy

Circular dichroism (CD) spectra were recorded using a JASCO J-715 CD-spectropolarimeter. The final spectra resulted from taking an average of five measurements performed at a temperature of 20°C (the sample cell temperature was controlled with a Peltier Type Temperature Control System), a scanning speed of 20 nm/min, data resolution of 1.0 nm and band width 1.0 nm. Measurements were carried out in a 1 mm path-length cuvette using DmChd64 and TcChd64 proteins at a concentration of 10 µM. The spectra were collected in a spectral range of 190–260 nm in buffer G. All spectra were corrected for the contribution of the buffer and all measurements were converted to molar residual ellipticity units [20]. The secondary structure content was calculated using CDPro spectra deconvolution software developed by Sreerama and Woody with IBasis 4, which is a reference set of 43 proteins. The means and standard deviations were calculated for the results obtained from three algorithms: CDSSTR, SELCON3 and CONTINLL [21].

### Analytical Size-exclusion Chromatography

Analytical size-exclusion chromatography was performed on a Superdex 200 10/300 GL (Amersham Biosciences) column connected to the ÄKTAexplorer (Amersham Biosciences) system and operated at 0.5 ml/min at room temperature. The column was equilibrated with buffer G and calibrated with a mixture of the following standard proteins: thyroglobulin (85 Å) [22], apoferritin (67 Å) [23], bovine serum albumin (35.5 Å) [22], ovalbumin (30,5 Å) [22], chymotrypsinogen (20.9 Å) [22], myoglobin (20.2 Å) [24] and cytochrome *c* (17 Å) [24]. Purified DmChd64 and TcChd64 in a 1 mg/ml concentration were loaded into an injection volume of 0.1 ml. The column volume (V_t_) was 24 ml and the column void volume (V_0_) of 7.83 ml was determined using blue dextran. The elution volume of each protein was used to calculate the gel phase distribution coefficients (K_AV_ factors) according to the equation: K_AV_ = (V_e_–V_0_)/(V_t_–V_0_) [25]. The Stokes radius (R_s_) values plotted against the corresponding K_AV_ factors of each standard protein were fitted to the standard curve, and then the R_s_ values for DmChd64 and TcChd64 were estimated.

### Analytical Ultracentrifugation

Sedimentation velocity (SV) experiments were performed on ProteomeLab XL-I Analytical Ultracentrifuge (Beckman Coulter) equipped with an AN-60 Ti 4-hole rotor, 12 mm path-length charcoal-filled double sector epons, sapphire windows and both absorbance and interference optics. Experiments were carried out at 20°C, at a rotor speed of 35 000 rpm using 400 µl of DmChd64 at 0.42 and 0.53 mg/ml and TcChd64 at concentrations of 0.35 mg/ml and 0.60 mg/ml in buffer G. To obtain high-quality fringes, the laser delay was adjusted prior to the runs. A series of 400 scans were obtained at 1.5 min intervals. The collected data were analysed with the size-distribution *c(s)* model implemented in SEDFIT version 14.1 software [26], [27]. Buffer density (*ρ* = 1.0108 g/cm^3^) and viscosity (*η* = 0.010307 mPa×s) were calculated from the composition of the buffer using SEDNTERP software available at: http://sednterp.unh.edu/ [28]. A partial, specific volume of 0.7254 and 0.729 ml/g at 20°C for DmChd64 and TcChd64, respectively, was calculated from the amino acid sequence using SEDNTERP [27].

### Small Angle X-ray Scattering

Small angle X-ray scattering (SAXS) experiments were performed at beamline P12 of the Petra III storage ring at DESY (Deutsches Elektronen-Synchrotron) in Hamburg. 20 µl of 5 mg/ml protein samples and corresponding matching buffers were loaded into a 96-well plate. Automated loading of the SAXS samples into the sample cuvette was achieved using a Hamilton syringe robot as described by Hura *et al*. 2009 [29]. All data collections were performed at 15°C. Integration, scaling, and buffer subtraction were accomplished using the Primus program [30]. SAXS data were collected over the s range of 0.0088–5 nm^−1^. Overlays of the merged data sets were used to detect concentration-dependent scattering in the lowest s region. Indirect Fourier transforms of the SAXS scattering curve were performed with GNOM [31]. The determination of a well-folded particle was made using a Kratky plot [32]–[34].

### Determination of Free Sulfhydryls [35]

Buffer G of purified DmChd64 and TcChd64 was exchanged using an Amicon Ultra-4 Centrifugal Filter Unit (Millipore) for 100 mM Na_2_HPO_4_, 150 mM NaCl, 2 mM EDTA, pH 8.0 and a protein concentration adjusted to 12 µM. A set of acetylcysteine standard solutions ranging from 10 µM to 100 µM in the reaction buffer (see above) was prepared. 2.5 µl of 10 mM5,5′-dithiobis-2-nitrobenzoic acid (DTNB) (Sigma Aldrich) [35] was added to 60 µl of each sample and absorbance at 412 nm [36] was measured after 15 minutes incubation using aV-630 UV-Vis Spectrophotometr (JASCO). The values obtained for the standards were plotted to generate a standard curve, and then the amounts of free sulfhydryl groups of DmChd64 and TcChd64 were estimated. For denatured proteins, the same procedure was repeated in a 100 mM Na_2_HPO_4_, 150 mM NaCl, 2 mM EDTA, 4% SDS, pH 8.0 buffer.

### In silico Analysis

The Uversky plot, and VSL2 calculations were made using PONDR at http://www.pondr.com [37]–[39]. Amino acid composition analysis was performed using the Composition Profiler at http://www.cprofiler.org [40], [41]. IUPred was performed at http://iupred.enzim.hu [42], [43]. BLAST [44] at http://blast.ncbi.nlm.nih.gov was used to compare amino acid sequences. The search for conserved domains was done by Pfam at http://pfam.sanger.ac.uk [45]. The prediction for tertiary structures in DmChd64 and TcChd64 was performed using the I-TASSER server available at http://zhanglab.ccmb.med.umich.edu/I-TASSER. ANCHOR at http://anchor.enzim.hu/was used for search for binding regions [46], [47]. All analyses were performed at default settings.

## Results

### *In silico* Analysis and Purification of DmChd64 and TcChd64

**Figure 1:**
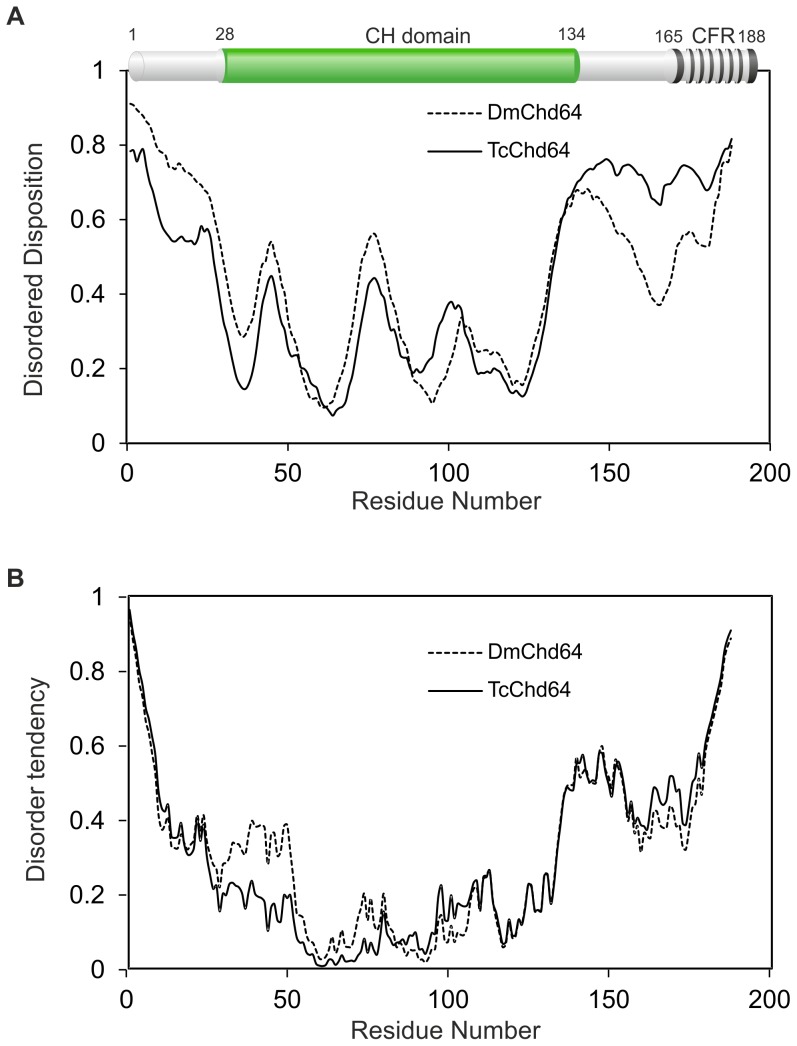
Computational analysis of DmChd64 and TcChd64 sequences. (A) and (B) The prediction of disordered regions from amino acid sequences. The prediction of the degree of disorder in DmChd64 (dashed line) and TcChd64 (solid line) was calculated from their primary structure using PONDR VSL2[37]–[39] (panel A) and IUPred [42], [43] (panel B) neural network predictors. For the PONDR and IUPred predictors, a score of more than 0.5 indicates a high probability of disorder. The top of the panel A represents a domain structure of Chd64 generated by Pfam [45]. Green colour represents calponin homology (CH) domain and black stripy region corresponds to calponin family repeat (CFR).

**Figure 2:**
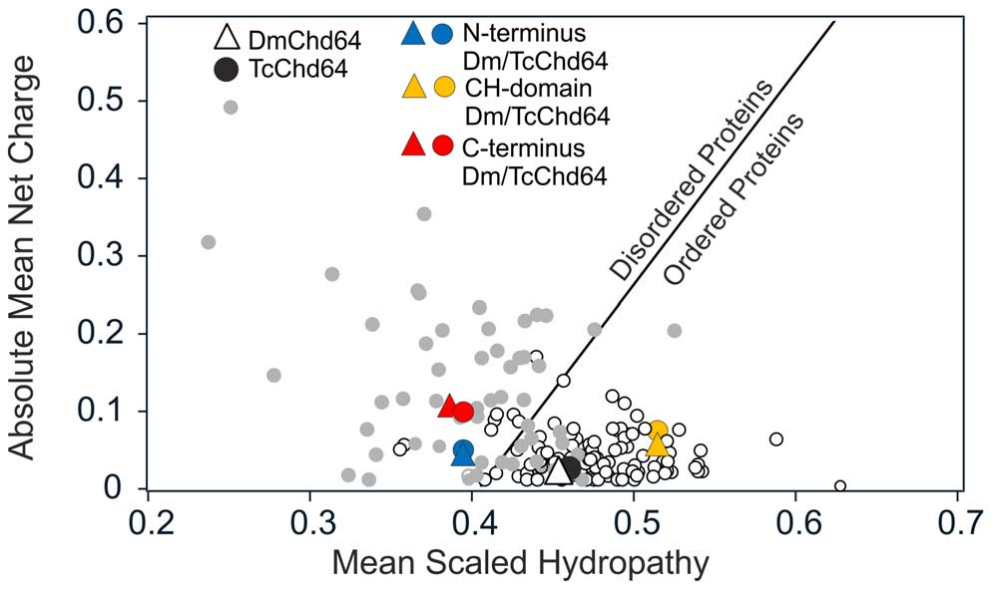
The Uversky plot of DmChd64 and TcChd64. The Uversky plot[37]–[39] divides proteins into globular (white dots) and IDP (grey dots) subsets based on their mean net charge versus mean hydropathy. The solid line represents permeable boundary between both subsets. DmChd64 is represented by triangles and TcChd64 by circles. The full-length DmChd64 protein is marked by a white triangle and TcChd64 by a black circle. For fragmented proteins, the following colors were used: blue for N-termini (1–30), yellow for CH domains (30–130) and red for C-termini (130–188).

In this work, *in silico* and *in vitro* analyses were performed to gain insight on the structure of DmChd64 and TcChd64. Prior to purification and *in vitro* analysis, *in silico* studies were conducted on DmChd64 and TcChd64 amino acids chains. BLAST [44] and Pfam [45] were used to compare amino acid sequences and identify conserved domains. Although the analysed proteins were from unrelated organisms – *D. melanogaster* belongs to *Diptera* while *T. castaneum* belongs to the *Coleoptera* order – they were found to have a high degree of homology, 74% of identity. The core of the proteins probably constitutes the CH domain, additionally C-terminal CFR was found (top of the Fig. 1A). The CH domain consisted mostly of α-helices [5], [48], thus the studied proteins should have a globular shape. *In silico* disorder predictors were applied in order to focus on the remaining fragments in the search for regions that could potentially be IDRs. PONDR VSL2 [37], [39] and IUPred [42], [43] were used to analyse the full-length proteins. As presented in Fig. 1A and 1B, there were no significant differences in the overall profiles obtained by the two predictors. Importantly, the core corresponding to the CH domain, residues 30–130, was predicted to be ordered, whereas the N- and C-terminal fragments were predicted to be disordered. The same tendency was observed when other predictors were applied (data not shown). The Composition Profiler [40], [41], a tool that provides statistics on the composition of analysed proteins against the distribution of amino acids of non-redundant proteins sequences in the SwissProt 51 database relative to the mean occurrence of disorder- and order-promoting residues present in IDPs (DisProt 3.4 database) and globular proteins (PDB S25 database) was used. The overall compositional profiles of DmChd64 and TcChd64 (Fig. S1A and S1B) indicated that the distribution of particular amino acids was similar what reflects their high homology. Neither of the outlines of the Chd64 proteins showed an obvious resemblance to globular or IDP profiles. Further, the Uversky diagram, a tool that plots mean net charge versus mean hydrophobicity and distinguish globular proteins from IDPs was used [28]. The boundary of the plot does not always clearly identify which subclass a protein belongs to. That was the case for the proteins under study. Full-length DmChd64 and TcChd64 were situated contiguously in a region occupied by globular proteins and IDPs (Fig. 2), thus the results were ambiguous. For that reason, a separate analysis was performed for regions corresponding to: the N-terminus (1–30), the CH domain (30–130) and the C-terminus (130–188). As presented in Fig. 2, the CH domain was located in the region occupied by globular proteins, whereas both terminal fragments were on the other side of the line where IDPs are found. Taken together, all the *in silico* results suggest that Chd64 proteins have a dual nature, i.e. a well-ordered core which probably corresponds to the CH domain and disordered terminal fragments.

**Figure 3:**
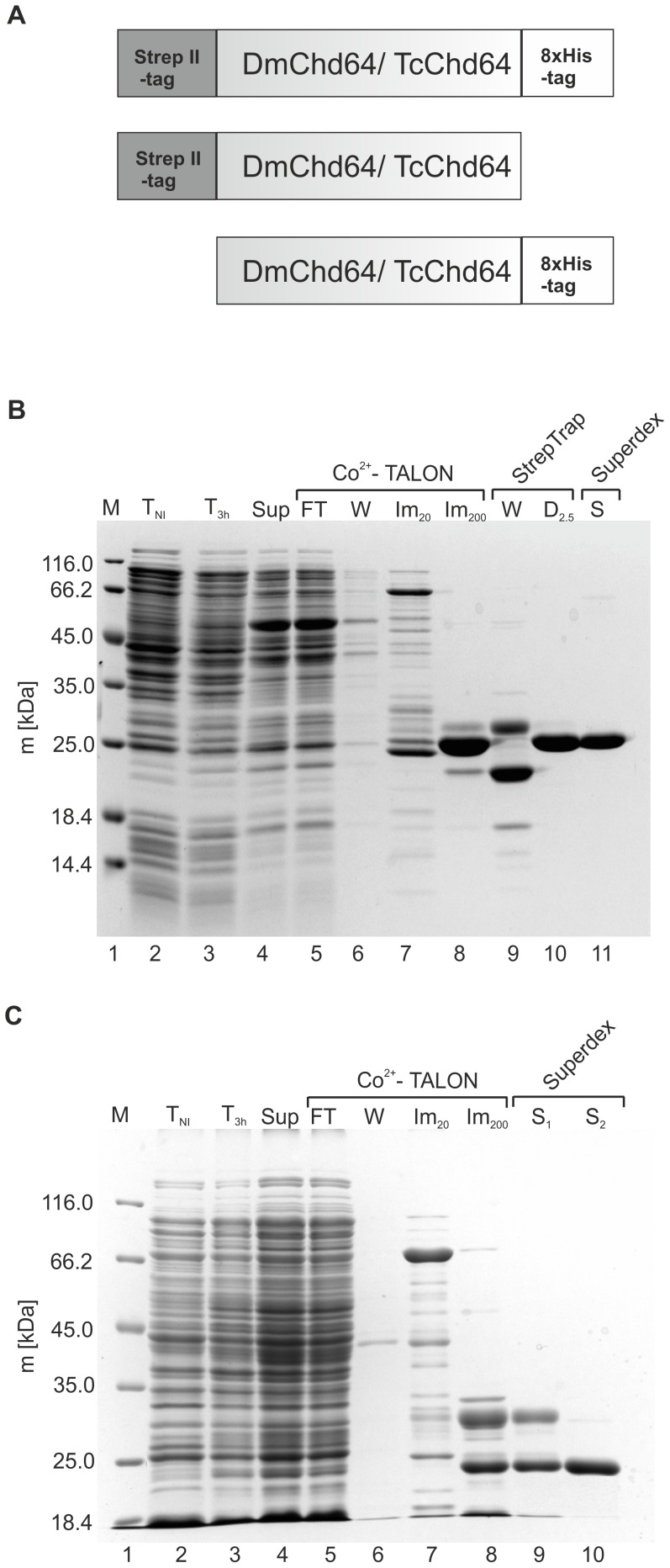
Schematic illustration and purification of recombinant DmChd64 and TcChd64. (A) Schematic illustration of recombinant DmChd64 and TcChd64. Recombinant constructs for DmChd64 and TcChd64 were prepared. DmChd64/TcChd64was N-terminally tagged with the Strep II-tag, and C-terminally tagged with the 8×His-tag. DmChd64/TcChd64 was N-terminally tagged with the Strep II-tag and DmChd64/TcChd64 was C-terminally tagged with the 8×His-tag. (B) Coomasie Brilliant Blue R-250-stained SDS-PAGE analysis of the expression and purification procedure of DmChd64. Lane 1, molecular mass standards; lane 2 and 3, whole cell extracts from bacterial cells containing the pQE80L-SXH plasmid with the cDNA insert (pQE80L-SXH/DmChd64) harvested before (T_NI_) and 3 h after expression induction with IPTG (T_3 h_); lane 4, soluble protein fractions obtained after cell lysis (Sup); lane 5, the flow-through (FT) fraction obtained from the Co^2+^-TALON column; lane 6, protein fractions containing impurities washed (W) from the Co^2+^-TALON column using buffer A; lane 7, protein fractions containing impurities eluted during the imidazole gradient sub-step using buffer A and B solutions containing 20 mM imidazole (Im_20_); lane 8, pooled fractions eluted from Co^2+^-TALON resin with buffer B containing 200 mM imidazole (Im_200_); lane 9, protein fractions containing impurities washed (W) from the Strep-Trap HP column using buffer C; lane 10, pooled fractions eluted from the Strep-TrapHP column with buffer D containing 2.5 mM desthiobiotin (D_2.5_); lane 11, pooled fractions of DmChd64 from the Superdex 200 10/300 GL column (S). (C) Coomasie Brilliant Blue R-250-stained SDS-PAGE analysis of the expression and purification procedure of TcChd64. Lane 1, molecular mass standards; lane 2 and 3, whole cell extracts from bacterial cells containing the pQE80L-XH plasmid with the cDNA insert (pQE80L-XH/TcChd64) harvested before (T_NI_) and 3 h after expression induction with IPTG (T_3 h_); lane 4, soluble protein fractions obtained after cell lysis (Sup); lane 5, the flow-through (FT) fraction obtained from the Co^2+^-TALON column; lane 6, protein fractions containing impurities washed (W) from the Co^2+^-TALON column using buffer E; lane 7, protein fractions containing impurities eluted during the imidazole gradient sub-step using buffer E and F solutions containing 20 mM imidazole (Im_20_); lane 8, pooled fractions eluted from the Co^2+^-TALON resin with buffer F containing 200 mM imidazole (Im_200_); lane 9, protein fractions containing impurities from the first peak from tandem connected Superdex 75 10/300 GL columns (S_1_)_;_ lane 10, pooled fractions of TcChd64 from the two tandem Superdex 75 10/300 GL columns (S_2_).

Expression and purification protocols were developed and optimized to experimentally examine the accuracy of the obtained bioinformatics results and to further characterize the biochemical properties of DmChd64 and TcChd64. In order to express DmChd64 and TcChd64 in the *Escherichia coli* BL21(DE3)pLysS strain, recombinant plasmid vectors were prepared containing cDNA coding for the target protein in fusion with: the Strep II-tag on the N-terminus, the 8×His-tag on the C-terminus and both of the tags (Fig. 3A). Expression of the target proteins was induced by IPTG for 3 h at 29°C and 182 rpm. All expressed recombinants were found to remain in a soluble fraction of bacterial proteins (data not shown). Initially, an attempt was made to purify DmChd64 with the 8×His-tag on the C-end. Although a range of conditions were applied, e.g. different pH and ionic strength at all stages of purification, it was not possible to obtain a homogenous protein sample. Every sample was contaminated with protein impurities and degradants (data not shown). Only a two-step purification of the DmChd64 derivative embodied with two tags, i.e. the Strep II-tag on the N-terminus and the 8×His-tag on the C-terminus, provided an efficient and homogenous sample. First, a soluble fraction of bacterial proteins (Fig. 3B, lane 4) was separated using a Co^2+^-Talon resin. The fraction eluted with 200 mM imidazole (Fig. 3B, lane 8) was then separated on a pre-packed StrepTrap HP column and proteins were eluted with 2.5 mM desthiobiotin using buffer D (Fig. 3B, lane10). At this stage, the sample was pure and gel filtration was employed to exchange buffer D with buffer G (Fig. 3B lane 11). The final protein sample was estimated to be over 95% pure, as judged from densitometric analysis (data not shown). In contrast to DmChd64, it was possible to use a simpler purification procedure for TcChd4. The C-terminally modified protein with the 8×His-tag was first separated on a Co^2+^-Talon resin. As revealed on SDS-PAGE, the fraction eluted with 200 mM imidazole consisted of two protein populations of different masses and electrophoretical mobility (Fig. 3C, lane 8). The fraction containing TcChd64 (Fig. 3C, lane 10) was separated from impurities (Fig. 3C, lane 9) on two tandem columns pre-packed with a Superdex 75 gel filtration medium. Densitometry analysis found the sample of TcChd64 to be over 94% pure (data not shown). To check the identity of the obtained proteins, the molecular weight values were determined by means of ESI mass spectrometry. Experimental values (DmChd64 = 23321.8±2 Da; TcChd64 = 22126.0±2 Da) were in compliance with theoretical values (DmChd64 = 23322.1 Da; TcChd64 = 22127.0 Da) for full-length proteins, with the exception of the first, N-terminal methionine. Cleavage of the N-terminal methionine is documented for recombinant proteins expressed in bacterial cells [49]. Additionally, the apparent molecular mass was determined using SDS-PAGE. Based on the migration pattern of the Unstained Protein Molecular Marker (Fermentas), DmChd64 was found to have migrated as its globular equivalent of 27470.0±0.5 Da (theoretical molecular mass 23322.1 Da), and TcChd64 migrated as a globular equivalent of 23600.0±0.6 Da (theoretical molecular mass 22127.0 Da). The observed discrepancy between the theoretical and experimental (SDS-PAGE based) molecular mass values is associated with the unusual amino acid composition of IDPs/IDRs which bind less SDS [50]. All values of molecular masses (experimental and theoretical) were estimated for tagged proteins.

### Circular Dichroism Analysis of DmChd64 and TcChd64

**Figure 4:**
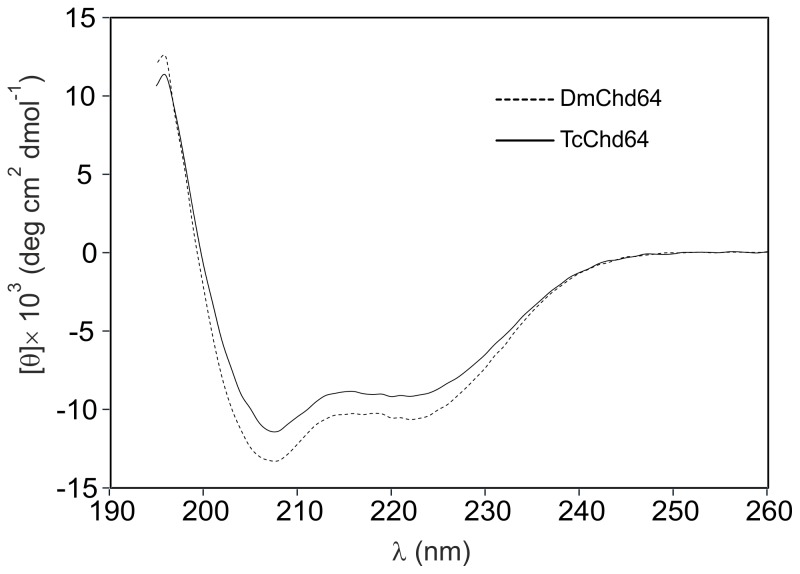
CD spectra of Chd64 from *D. melanogaster* and *T. castaneum*. CD spectra of DmChd64 (dashed line) and TcChd64 (solid line). The far-UV spectra were recorded in 50 mM Na_2_HPO4, 150 mM NaCl, pH 7 at room temperature. For every measurement, 10 µM of protein (223 µg/ml for DmChd64 and 222 µg/ml for TcChd64) was used. Both spectra of the native proteins display deep minima around 222 nm and 208 nm, which is characteristic of an ordered conformation.

CD spectroscopy was used for analysis, since it enables detection of all secondary structure types found in proteins from the characteristic CD spectra profiles in the far UV [51]. In accordance with bioinformatics predictions (see above), the obtained spectra were characteristic for proteins that are mostly ordered [20]. As presented in Fig. 4, both proteins display deep minima around 208 nm and 222 nm, indications of an ordered secondary structure [20], [52]. Again, although the studied proteins had a high degree of homology, some differences in secondary structure were noticeable. In order to obtain more detailed numerical data on the impact of secondary structures, CDPro deconvolution software was used with CDSSTR, SELCON3 and CONTINLL algorithms [21]. Results obtained with IBasis 4 were averaged and presented in Table 1. The analysis revealed that α-helices were the most prevalent type of secondary structure in both DmChd64 and TcChd64 with values of 39.3±1.2% and 34.4±1.7%, respectively. The predominance of α-helices is probably due to the presence of the CH domain, which has a mostly helical fold [48]. The β-strands were the least prevalent and there were slightly more in TcChd64 (15.7±1.5%) than in DmChd64 (11.2±1.6%). Importantly, although the spectra lines with deep minima around 208 nm and 222 nm resembled the profiles of ordered proteins, detailed computational analysis revealed that nearly 30% of the polypeptide chain is formed by disordered regions. This is consistent with the preliminary *in silico* analysis.

**Table 1:** Estimation of the DmChd64 and the TcChd64 Secondary Structure Content from CD Spectra.

Protein	α-Helix (%)			β-Strand (%)			Other (%)	
	Regular	Distorted	Total	Regular	Distorted	Total	Turns (%)	Unordered (%)
DmChd64	21.6±0.8	17.7±0.4	39.3±1.2	5.1±1.4	6.1±0.3	11.2±1.6	21.0±1.5	27.7±1.0
TcChd64	18.8±1.2	15.6±0.8	34.4±1.7	8.6±1.1	7.1±0.4	15.7±1.5	20.5±1.4	28.6±0.1

The secondary structure content was calculated using CDPro spectra deconvolution software with IBasis 4 which is a reference set of 43 proteins. The means and ± standard deviation were calculated for results obtained from three algorithms: CDSSTR, SELCON3 and CONTINLL.

### Hydrodynamic Properties of DmChd64 and TcChd64

**Figure 5:**
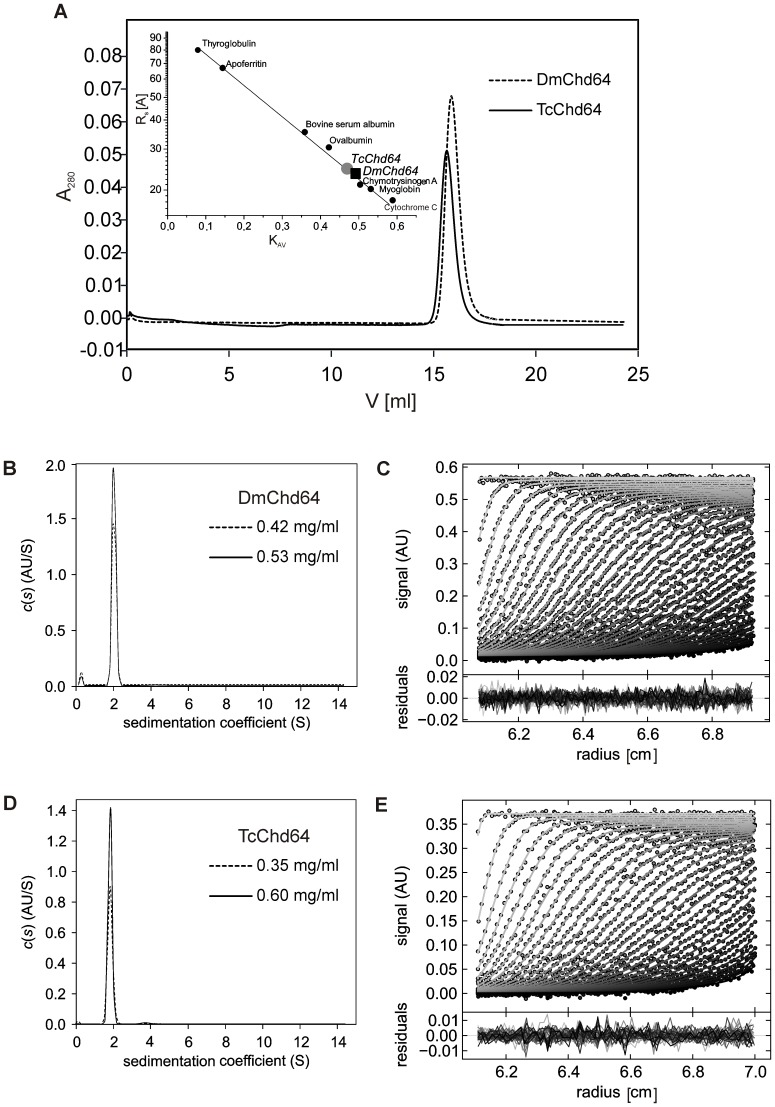
Hydrodynamic properties of DmChd64 and TcChd64. (A) Analytical size-exclusion chromatography of DmChd64 (dashed line) and TcChd64 (solid lane). Experiments were performed on a Superdex 200 10/300 GL column equilibrated with 50 mM Na_2_HPO4, 150 mM NaCl, pH 7 at room temperature and at a flow rate of 0.5 ml/min; an injection volume of 0.1 ml was used and the protein concentration was 1 mg/ml. The inset shows the calibration curve determined using standard proteins (black dots). The black square corresponds to DmChd64 and the grey dot to TcChd64. (B), (D) Sedimentation velocity analytical ultracentrifugation analysis. Superposition of the distribution of sedimentation coefficients, c(s) derived via SEDFIT from SV data for DmChd64 in two different concentrations (B) and for TcChd64 in two different concentrations (D) measured using absorbance optics at 280 nm during an SV experiment at 30 000 rpm at 20°C, standardised to water at 20°C. (C), (E) Example of the sedimentation profile of DmChd64 at 0.53 mg/ml (C) and TcChd64 at 0.6 mg/ml (E). Superposition of selected experimental (circles) and fitted (solid lines) SV profiles corrected for all systematic noises, with an rmsd of 0.004531 for DmChd64 and 0.003496 for TcChd64 indicating a good fit of the SV data. The inset shows the superposition of the residuals between the experimental and fitted curves.

Analytical size exclusion chromatography and analytical ultracentrifugation were applied to determine basic shape-related hydrodynamic parameters such as Rs [25] and the frictional coefficient (f_0_/f_min_) and thus gain insight into the structure of DmChd64 and TcChd64. Based on the results obtained from analytical size-exclusion chromatography (Fig. 5A), the experimentally determined Rs for both proteins were found to be larger than the theoretical ones; the differences were 1.2 Å for DmCh64 and 2.6Å for TcChd64 (Table 2). The same tendency was observed for samples in three different concentrations (data not shown), which suggests that DmChd64 and TcChd64 occur as monomers without oligomerization potential. The two proteins appear to have a relatively loose structure, since the molecular volume is larger compared to totally globular equivalents of the same theoretical mass (Table 2). The analytical ultracentrifugation results (Fig. 5B–E) also showed that the studied proteins cannot be classified as entirely globular. The f_0_/f_min_ coefficient, an indicator of hydrodynamic shape, is determined from the function of the size and shape of the molecule. The values were 1.54±0.005 and 1.52±0.000 for DmChd64 and TcChd64, respectively (Table 3), which provides evidence that the proteins have an elongated, ellipsoidal, not spherical shape (see Discussion). This elongation may be caused by the existence of IDRs at both ends as predicted by PONDR and IUPred (Fig. 1A, 1B). Additionally, the values of the sedimentation coefficient (S_w20_) were constant for various concentrations (Table 3), thus the determined properties are not concentration-dependent (Fig. 5B, 5D). Moreover, the results from every concentration of the analysed proteins yielded a very good fit, judging from the low rmsd values (Table 3, Fig. 5C, 5E).

**Table 2:** Hydrodynamic Properties of the DmChd64 and the TcChd64.

Protein	MW [Da]	R_s_ [Å]		*V_s_*×10^3^[Å^3^]		*ρ*[*Da*/Å^3^]	
		Theor^a^	Exp	Theor^b^	Exp^c^	Theor^b^	Exp^c^
DmChd64	23322.1	22.2	23.4±0.1	45.8	53.7	0.51	0.43
TcChd64	22127.0	21.7	24.3±0.2	42.8	60.1	0.52	0.37

a Determined from the equation: log (R_s_^N^) = (0.8054±0.031)+(0.3949±0.016)×log (MW) for native globular proteins.

b Calculated using theoretical R_s_.

c Calculated using experimental R_s_.

**Table 3:** Hydrodynamic Properties of the DmChd64 and the TcChd64. Analytical ultracentrifugation.

Protein	Concentration (mg/ml)	rmsd	f/f_min_	s_20,w_ (S)	%^1^	R_S_ [Å]	MW [Da]
DmChd64	0.42	0.00551	1.55	1.974	96.2	29.8	24 544
	0.53	0.00453	1.54	1.968	96.0	29.7	24 398
TcChd64	0.35	0.00402	1.52	1.936	97.1	28.8	23 385
	0.60	0.00350	1.52	1.903	98.6	28.5	22 761

1 Are given considering 100% for the all types of species.

### Small Angle X-ray Scattering (SAXS)

**Figure 6:**
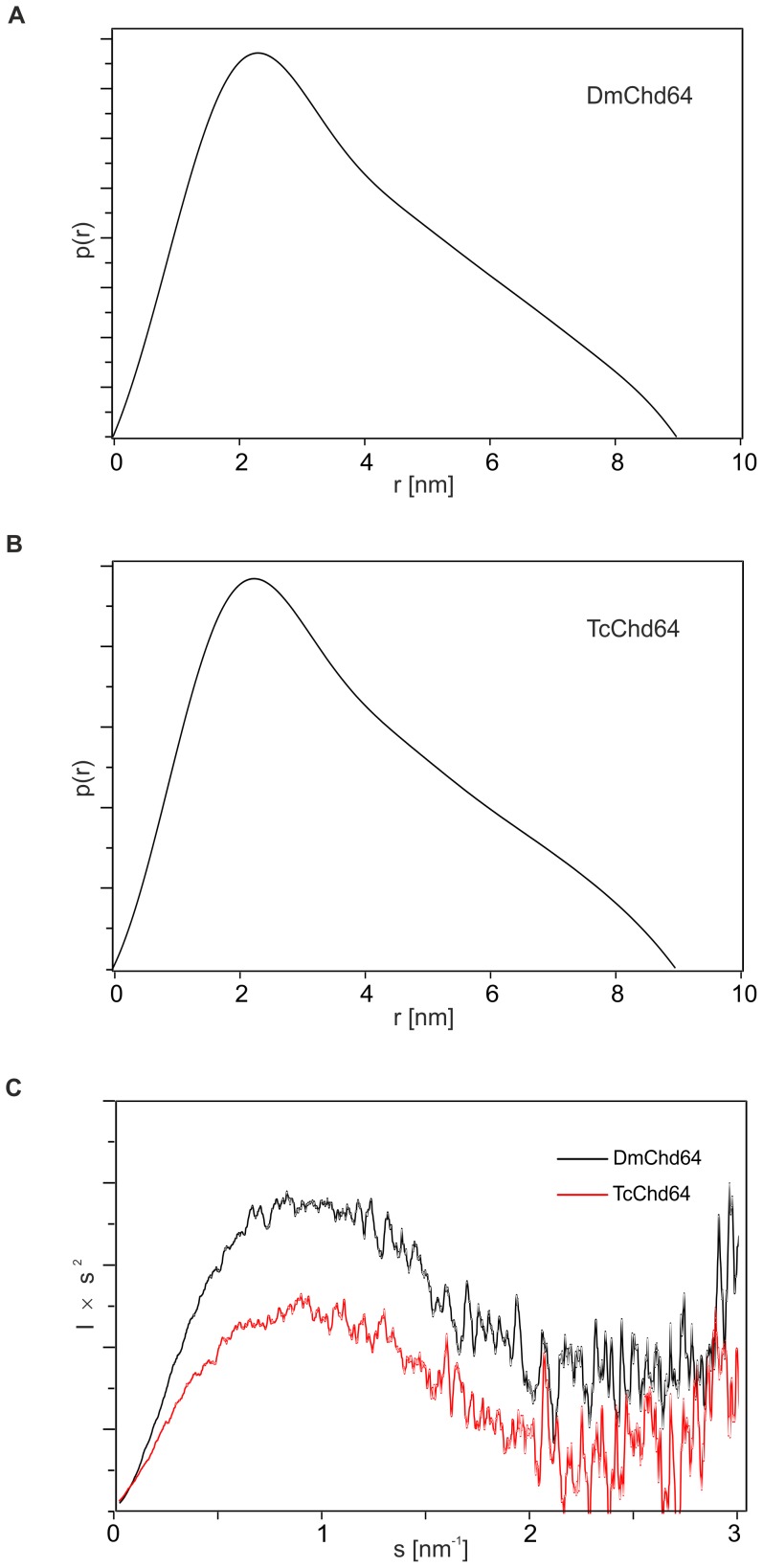
Small Angle X-ray Scattering characteristics for DmChd64 and TcChd64. (A), (B) Pair distribution function p(r) of DmChd64 (A) and TcChd64 (B). For both analysed proteins p(r) functions are asymmetric, which indicates the proteins have an elongated shape. (C) Kratky plot analysis of DmChd64 (black line) and TcChd64 (red line), where the intensity of scattering is plotted as Is^2^ versus s. For both proteins, the plots exhibit one maximum. At higher s values the curves reach a plateau, which suggests the existence of disorder.

SAXS was used to complement the previous analysis and obtain further insight into the structure of Chd64. SAXS is a powerful technique particularly well adapted to the study of flexible, less compact or even extended macromolecules in solution [53]. The pair distribution (p(r)) function is a histogram of all the interatomic distances (r) within the proteins, providing information on the size and overall shape of the molecules [54]. The histogram shows mean particle size from the radius of gyration (R_g_) and maximal intramolecular distance (D_max_), two parameters that show the degree of compactness of the molecules. Additionally, the D_max_ parameter calculates the maximal degree of extension reached by the molecule in solution [33]. The obtained p(r) function was asymmetric; the value of D_max_ was 8.9 nm for DmChd64 and 9.0 nm for TcChd64 and R_g_ values were 2.95 nm and 2.93 for DmChd64 and TcChd64, respectively (Fig. 6A and 6B). The shape of the histogram and the high values of D_max_ indicate that the proteins have an elongated shape.

Next, the volume of the molecules was determined. Based on Porod’s equation(29), the experimental hydrated particle volume of the studied proteins was 61.28 nm^3^ for DmChd64 and 63.47 nm^3^ for TcChd64, while the calculated theoretical volume was 28.38 nm^3^and 26.64 nm^3^for DmChd64 and TcChd64, respectively. The significant differences between the theoretical and experimental volumes were caused by the low density of the molecules, which suggests the existence of flexible, disordered regions [54], [55]. IDRs can adopt an extended conformation and this would account for the increase in size and low density of the molecules compared to the globular equivalents.

The Kratky plot is an extremely useful representation of scattering intensity, providing a quick assessment of protein structure [32]. The profiles of the Kratky plots for both proteins are presented in Fig. 6C. The Kratky plot is the scattering pattern of Is^2^ versus s. The scattering intensity of a globular protein with a well-defined, solvent-accessible surface follows the Porod law and decreases as s^4^ in the large s region. The corresponding Kratky plot for a globular protein exhibits a typical bell-shape with a well-defined maximum [50]. Conversely, for a random coil, the scattering intensity has a limiting behavior of s^2^ at high s, as indicated by the Debye law. Therefore, the Kratky plot of a fully unfolded protein will not show a clear maximum but will exhibit a plateau in the high s region, sometimes followed by a higher level as s increases, depending on the local rigidity of the chain [54], [55]. For proteins with highly dynamic fragments attached to globular particles, a dual behavior can be seen on the Kratky plot. The plot possesses one maximum which corresponds to an ordered part but at higher s values the curve reaches a plateau caused by disordered fragments [50], [56]. This was the case for DmChd64 and TcChd64. As presented on Fig. 6C, the analysed profile had one bump at s≈0.9 nm^−1^ followed by a plateau for s>1.7 nm^−1^.

Altogether, the hydrodynamic properties of Chd64 as observed from gel filtration, analytical ultracentrifugation and the SAXS (p(r) function), indicate that the analysed proteins have an asymmetric, elongated shape. The molecules are of low density, which may stem from the existence of a loose, non-globular conformation. Finally, the Kratky plot revealed the presence of a disordered structure in Chd64.

### Determination of Free Sulfhydryls in DmChd64 and TcChd64

**Figure 7:**
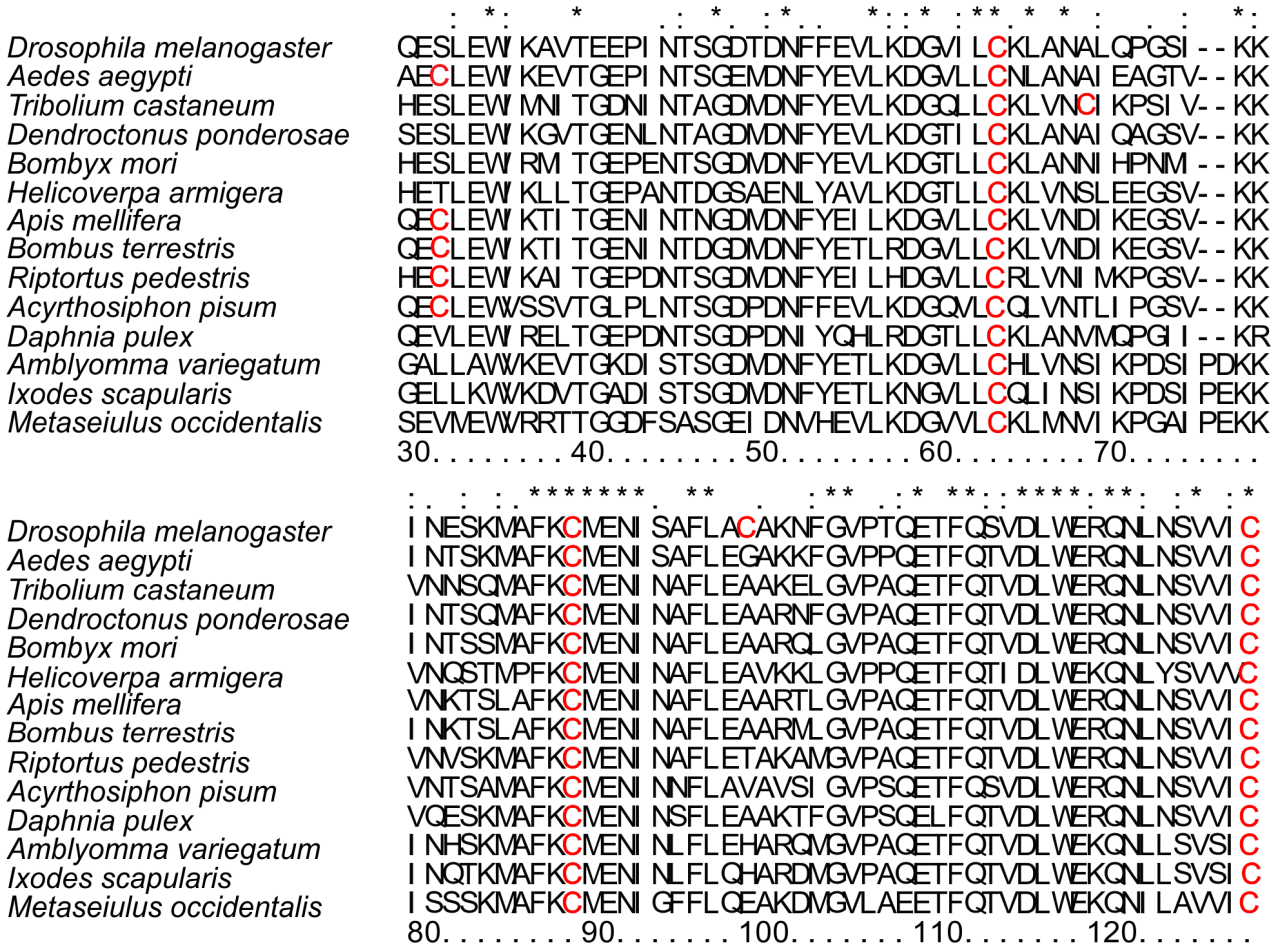
Amino acid sequence alignment of proteins containing a CH domain. The figure represents the sequence alignment for two randomly chosen representatives of five different insect orders, and four additional proteins aligned from non-insect invertebrates: *Drosophila melanogaster* and *Aedes aegypti* from Diptera; *Tribolium castaneum* and *Dendroctonus ponderosae* from Coleptera; *Bombyx mori* and *Helicoverpa armigera* from Lepidoptera; *Apis mellifera* and *Bombus terrestris* from Hymenoptera; *Riptorus pedestris* and *Acyrthosiphon pisum* from Hemiptera; *Daphnia pulex*, *Amblyomma variegatum*, *Ixodes scalpularis* and *Metaseiulus occidentalis* from non-insect invertebrates. Stars indicate positions which have a single, fully-conserved residue; colons indicate that one of the following strong groups is fully conserved; dots indicate that one of the following weaker groups is fully conserved. C residues are highlighted in red. The numbering of the residues corresponding to the *D. melanogaster* Chd64 sequence is given at the bottom of the alignment. Sequences were taken from UniProtKB (http://www.uniprot.org/). All alignments were done using ClustalIX.

Since DmChd64 and TcChd64 show 74% identity of the primary structure, we decided to check which residues share the same position. ClustalIX, a multi-sequence alignment program was used to align both Chd64 proteins with randomly chosen representatives of a distinct organism. The alignment of amino acid sequences from five orders of insects and several non-insect invertebrate species is presented in Fig. 7. Interestingly, every randomly chosen species possesses four C residues and three of those are in the same position. This pattern seems to be universal for most, if not all invertebrates. Subsequently, a quantitative analysis was performed of free thiols using the Ellman assay to investigate the reactivity of C residues to shed additional light on the structural features of DmChd64 and TcChd64. Only exposed, non-modified C residues which had access to the reagent show reactivity [35]. In other words, the reactivity of the C residues reflected their position within the protein conformers. The Ellman reagent DTNB reacts rapidly with free thiols yielding a mixed disulfide and 2-nitro-5-thiobenzoic acid (TNB), the concentration of which can be measured at 412 nm [35], [36]. DmChd64 and TcChd64 were incubated with DTNB for 15 min at room temperature and absorbance was read using the V-630 UV-Vis Spectrophotometer (JASCO). The molar concentration of free thiols in DmChd64 was 44.4 µM ±0.6 per 11.52 µM of the protein sample what indicates existence of four reactive thiols in a single protein molecule. In contrast, TcChd64 had three reactive thiols, since a concentration of 37.2±0.5 µM was determined per 11.52 µM of the protein sample, meaning that one C residue in protein molecule was less reactive or less accessible to the DTNB. As a control, the assay was performed for SDS-denatured proteins at the same concentrations. The DmChd64 molar concentration of free thiols was found to be 44.9±0.7 and for TcChd64 it was 45.1±0.8. The observed quantitative differences in the reactivity of the thiols in non-denaturated proteins may have arisen from the specific location in the protein conformers and may reflect some structural differences.

**Figure 8:**
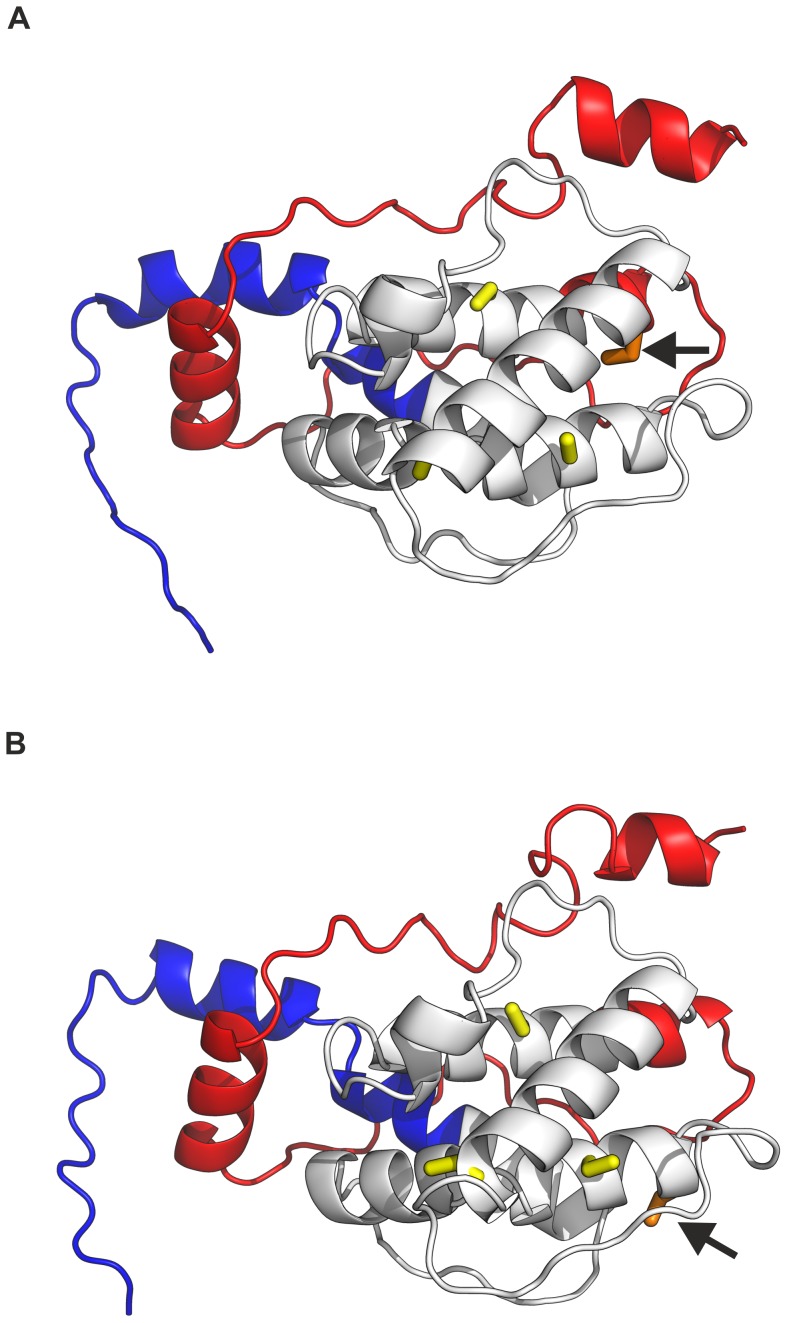
3D structure predictions of DmChd64 and TcChd64. The model of the DmChd64 structure is presented on panel (A) and TcChd64 on (B). Structure conformation was generated by web-based tool ITASSER [57], [58] and results were visualised by PyMOL [59]. N-terminal tails are highlighted in blue and C-terminals are in red. The CH domain is marked in grey, conserved C residues are yellow. The arrows point to non-conserved C residues marked in orange.

The online platform ITASSER was used to build a 3D model of DmChd64 and TcChd64, focusing on the position of C residues within the conformers. The program is known for providing relatively accurate models using state-of-the-art algorithms. Results were obtained for five models with different confidence scores (c-values) ranging from [−5, 2] [57], [58]. Models for the highest c-values were loaded into the PyMOL [59] molecular visualisation system and are shown on Fig. 8A and 8B. The models revealed that all four residues in both DmChd64 and TcChd64 were located in helical regions. The Ellman assay showed that for TcChd64 one C residue was less reactive. Unfortunately, it is not clear from the model which C residue had less access to DTNB (see Discussion). Nevertheless, the models illustrated the helical nature of the fragments which probably corresponded to the CH domain and IDRs on both termini of the protein molecules. This structural organisation was suggested by the *in silico* disorder predictors. *In vitro* analyses indicated that DmChd64 and TcChd64 had an elongated, asymmetric shape that may have been caused by the existence of IDRs on the protein termini. In fact, the models generated for both Chd64 proteins visualized all the structural characteristics described above.

## Discussion

This study presents the results of a series of *in silico* and *in vitro* analyses of the structural organisation of two calponin-like Chd64 proteins from *Drosophila melanogaster* and *Tribolium castaneum* in order to better understand the molecular basis of their role in the host organisms. A possible biological function of Chd64 from *D. melanogaster* has previously been proposed. The protein is believed to be one of the key parts of a multi-protein complex which regulates insect development by cross-linking hormonal signaling pathways from 20E and JH [3]. Derived from genome sequencing, Chd64 from *T. castaneum* is a putative, uncharacterised protein whose role has not yet been described. TcChd64 shows a 74% sequence identity with DmChd64, thus both proteins were expected to share similar structural features. Pfam [45] was used to search for conserved domains. A N-terminal CH domain and single, C-terminal CFR was found (Fig. 1A). The family of proteins that contain CH domains is comprised of three distinct classes which differ by the number of CH domains, the location of the domain within a sequence and the presence of additional domains, e.g. CFR, EF hand or ABD [60]. Most proteins which contain a CH domain are involved in actin cross-linking and bundling, whereas others are modulators of cellular signaling [5]. The Pfam [45] results assigned both DmChd64 and TcChd64 to a CH3 class characterised by the presence of a single, approximately 100-residue long CH domain. Proteins belonging to CH3-class can bind to cytoskeletal components, e.g. calponin or transgelin, other like Vav or IQGAP, are signal modulators. Following the search for conserved residues conducted by Stradal *et al.* [60], we assigned DmChd64 and TcChd64 to the transgelin-like subgroup. Transgelin is an abundant protein identified in many species and is a member of the calponin family of actin-binding proteins [61], however it has been also shown that the protein performs other biological, non-cytosceletal functions[62]–[64]. HaCal is an example of an invertebrate transgelin-like protein that functions as hormonal signal modulator in *Helicoverpa armigera* [4]. Many transgelin-like proteins are awaiting characterisation on a molecular level in order to understand their exact biological role.

*In silico* studies of DmChd64 and TcChd64 were conducted to identify the molecular organization of their primary structures. Since proteins participating in binding, regulation and signal transduction are often rich in IDRs [65], a range of predictors was applied to look for intrinsic disorder. The region identified by Pfam [45] as a CH domain showed a high probability of having a well-ordered structure, whereas remaining fragments appeared to be IDRs (Fig. 1A, 1B). Using the Uversky method [37], both full-length Chd64 proteins were found in a region occupied by folded proteins and IDPs. However, analysis of the protein fragments showed that the central region differed from the terminal regions in terms of the type of amino acid composition. The residues comprising the CH domain tended to have an ordered structure, whereas terminal regions consisted of residues whose mean net charge was relatively high and mean hydropathy relatively low, which would reduce the ability to form hydrogen bonds within a backbone and prevent the formation of a well-ordered structure (Fig. 2). In total, the *in silico* analyses suggested that the structure of DmChd64 and TcChd64 is a combination of ordered and disordered fragments.

CD spectroscopy was applied *in vitro* to evaluate the content of secondary structures. DmChd64 and TcChd64 were found to have a relatively high content of secondary structures. In particular, CDPro deconvolution software [21] predicted that the α-helix would be the most prominent secondary structure type and it accounted for over one third of both polypeptide chains. Importantly, nearly 30% of the polypeptide chains did not appear to form any defined secondary structure, which is consistent with the results from PONDR and IUPred (Fig. 1A, 1B). Visualisation of the protein structure by I-TASSER [57], [58] showed that the central region corresponding to the CH domain consisted of four main helices, three parallel and one perpendicular, representing a typical CH domain [48] with the remaining fragments being IDRs (Fig. 8). Furthermore, in each of five generated models, the central fragments showed minimal conformational differences in contrast to the terminal fragments, where differences were decidedly more prominent (data not shown). This might have been due to the loose, labile structure of IDRs. The presence of IDRs could also explain the aberrant electrophoretic mobility of DmChd64 and TcChd64. Both proteins migrate in an electric field that would be equivalent to proteins of higher molecular weight. This phenomenon is characteristic for IDPs and stems from their unusual amino acid composition. IDPs bind less SDS, decreasing their migration velocity and giving an apparent molecular weight that is higher than the real one calculated by mass spectrometry. Size exclusion chromatography [25] and ultracentrifugation [27], [28] were utilised to determine basic hydrodynamic properties correlating to the overall shape of the DmChd64 and TcChd64 molecules. Size exclusion chromatography revealed that the experimental Rs of both proteins was larger than the theoretical ones (Table 2). Again, this discrepancy may have been due to the presence of IDRs since disordered structures have larger hydrodynamic dimensions than entirely globular equivalents of the same molecular mass). The f_0_/f_min_ coefficient, an indicator of hydrodynamic shape, ranges from 1.2 for very globular to 1.8 for asymmetric proteins [27]. The f_0_/f_min_ determined for DmChd64 and TcChd64 was over 1.5 (Table 3), which is another indication that these molecules are largely globular but with an ellipsoidal rather than spherical shape. Sedimentation coefficient values (Table 3) for different concentrations of the studied proteins showed that the determined structural properties were not a function of the concentrations. This eliminates the possibility that the larger Rs and f_0_/f_min_ were due to oligomerisation. Additionally, the results obtained by SAXS determined that the proteins were elongated, since the p(r) function was asymmetrical (Fig. 6A, 6B). The molecules were characterised by low density, which suggests the presence of unstructured and flexible regions. This was further confirmed by the Kratky plot which was typical for proteins possessing a dual nature, i.e. a well ordered core and disordered regions (Fig. 6C). The N-terminal IDR of TcChd64 may explain the decrease in reactivity of the non-conserved cysteine in TcChd64. Results of the Ellman assay [35], [36] revealed four reactive C residues in DmChd64, whereas in TcChd64 three were reactive and one showed significantly less reactivity. In the model generated by I-TASSER [57], [58] (Fig. 8A, 8B) all the C residues were facing the outside of DmChd64 and TcChd64. Since the non-conserved C residue in TcChd64 was close to the edge of an α-helix and neighbored the C-terminal fragment that is an IDR, it is possible that this flexible tail serves as a veil, and consequently, the residue has lower access to DTNB.

In consideration of the results discussed above, the relevant question is how DmChd64 and TcChd64 could work as regulatory proteins as showed by Li *et al.* [3]. The function of the CH domain is not clear, and initially, the motif was believed to account for the actin binding properties of calponin-like proteins. Further studies showed, however, that a single CH is neither sufficient nor necessary for F-actin binding [10]. Furthermore, actin is highly conserved in all living organisms, thus so should its binding proteins be, yet there is such diversity among CH domains, that different classes have been distinguished [60]. A CH domain is present in other regulatory proteins, which suggests that at least some of the proteins that contain a CH domain have a distinct function. A presence of single CFR in Chd64 may indicate its cytoskeletal function, however a role in hormonal signal transduction of DmChd64 has also been shown [3]. Nevertheless the molecular bases of the multiprotein interaction remain unknown. An important aspect of the structural organization of the studied proteins may be the presence of IDRs on the protein termini, which could be responsible for interaction within a multi-protein complex. IDPs/IDRs provide a large interaction surface compared to globular proteins of similar length [12]. Their flexibility and the exposure of short linear peptides enable disordered proteins to interact with numerous and various partners, including other proteins, nucleic acids, membranes or small molecules [13]. Within probable C-terminal IDRs, ANCHOR [46], [47] predicted existence of potential protein binding regions (Fig. S2). IDPs/IDRs often fold upon binding with relatively high specificity and low affinity, thus they can rapidly associate and initial signaling process but when the process is finished they easily dissociate [65]. Disorderness is non-homogenously distributed within IDPs and is more often observed on protein termini than in the center of a polypeptide chain. The functional importance of multi-tasking disordered protein termini has been very recently revised by Uversky [66]. p53 is one of many classic examples of a protein that consists of a globular module with disordered termini. The protein has an ordered core flanked by N- and C-terminal IDRs. The presence of labile regions gives p53 the potential to perform multi-functional roles by enabling interaction with various partners [67]. Likewise, the IDRs of DmChd64 and TcChd64 with their flexible and dynamic nature could act as affinity tuners that reorganize multi-protein complex as proposed by Li *et al*. [3].

The results of our study demonstrate that DmChd64 and TcChd64 have a similar structural organisation. The proteins consist of a well-defined globular core which probably corresponds to a CH domain flanked by IDRs. The disordered nature of terminal regions would account for the elongation of the overall shape and give rise to specific hydrodynamic properties. The specific structural organization of DmChd64 and TcChd64 makes them perfect candidates for performing regulatory functions and further investigation on this is warranted.

## Supporting Information

Figure S1 ***In silico*** **analysis of DmChd64 and TcChd64 amino acid composition.** (A) and (B) present amino acid composition analysis of DmChd64 and TcChd64 (grey dotted bars), respectively, using the Composition Profiler [40]. Values above zero show the enrichment of amino acids in proteins and values below zero show the depletion of amino acids in relation to proteins from the SwissProt51 database. A comparison of the frequency of residues in IDPs from the DisProt 3.4 data set (black bars) and PDB S25 (grey bars) is also presented. The X axis is ordered by the flexibility of the amino acid residues according to the scale based on the B-factor of the backbone atoms, where the most rigid residues are on the left and the most flexible are on the right [37], [38].
(TIF)

Figure S2 **The prediction of protein binding regions in disordered proteins for DmChd64 and TcChd64 by ANCHOR.** The prediction of the probability of existence of protein binding regions in DmChd64 (dashed line) and TcChd64 (solid line) was calculated from their primary structure using ANCHOR [46], [47]. The regions with values above 0.5 are potential proteins’ binding regions.
(TIF)
