# Supplementary figures and images for: Calponin-Like Chd64 Is Partly Disordered
Source: PLoS One. 2014 May 7;9(5):e96809. doi: 10.1371/journal.pone.0096809 (PMC4013081; doi:10.1371/journal.pone.0096809)

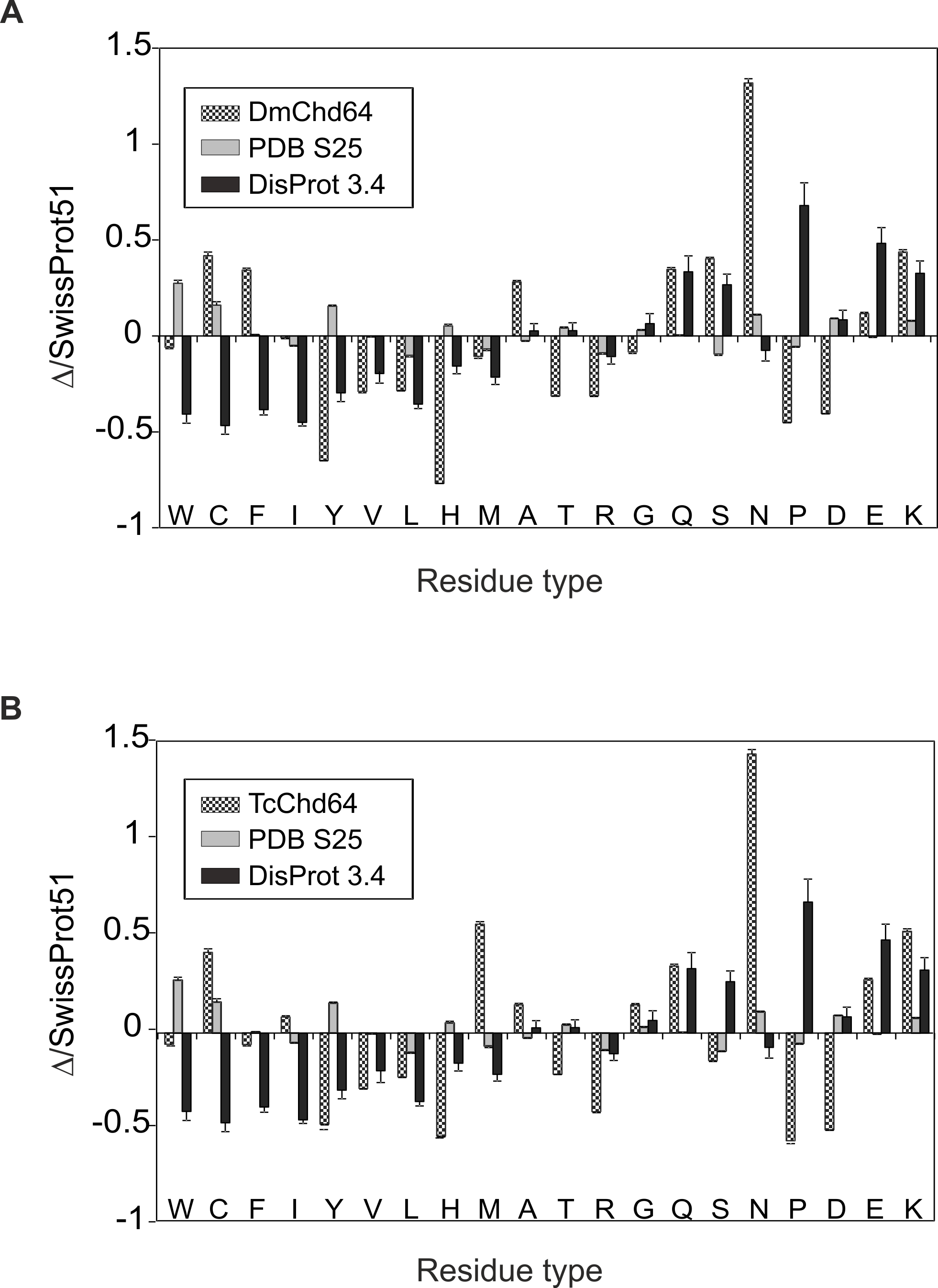

Supplement: Figure S1 — In silico analysis of DmChd64 and TcChd64 amino acid composition. (A) and (B) present amino acid composition analysis of DmChd64 and TcChd64 (grey dotted bars), respectively, using the Composition Profiler [40]. Values above zero show the enrichment of amino acids in proteins and values below zero show the depletion of amino acids in relation to proteins from the SwissProt51 database. A comparison of the frequency of residues in IDPs from the DisProt 3.4 data set (black bars) and PDB S25 (grey bars) is also presented. The X axis is ordered by the flexibility of the amino acid residues according to the scale based on the B-factor of the backbone atoms, where the most rigid residues are on the left and the most flexible are on the right [37], [38]. (TIF) [file pone.0096809.s001.tif]

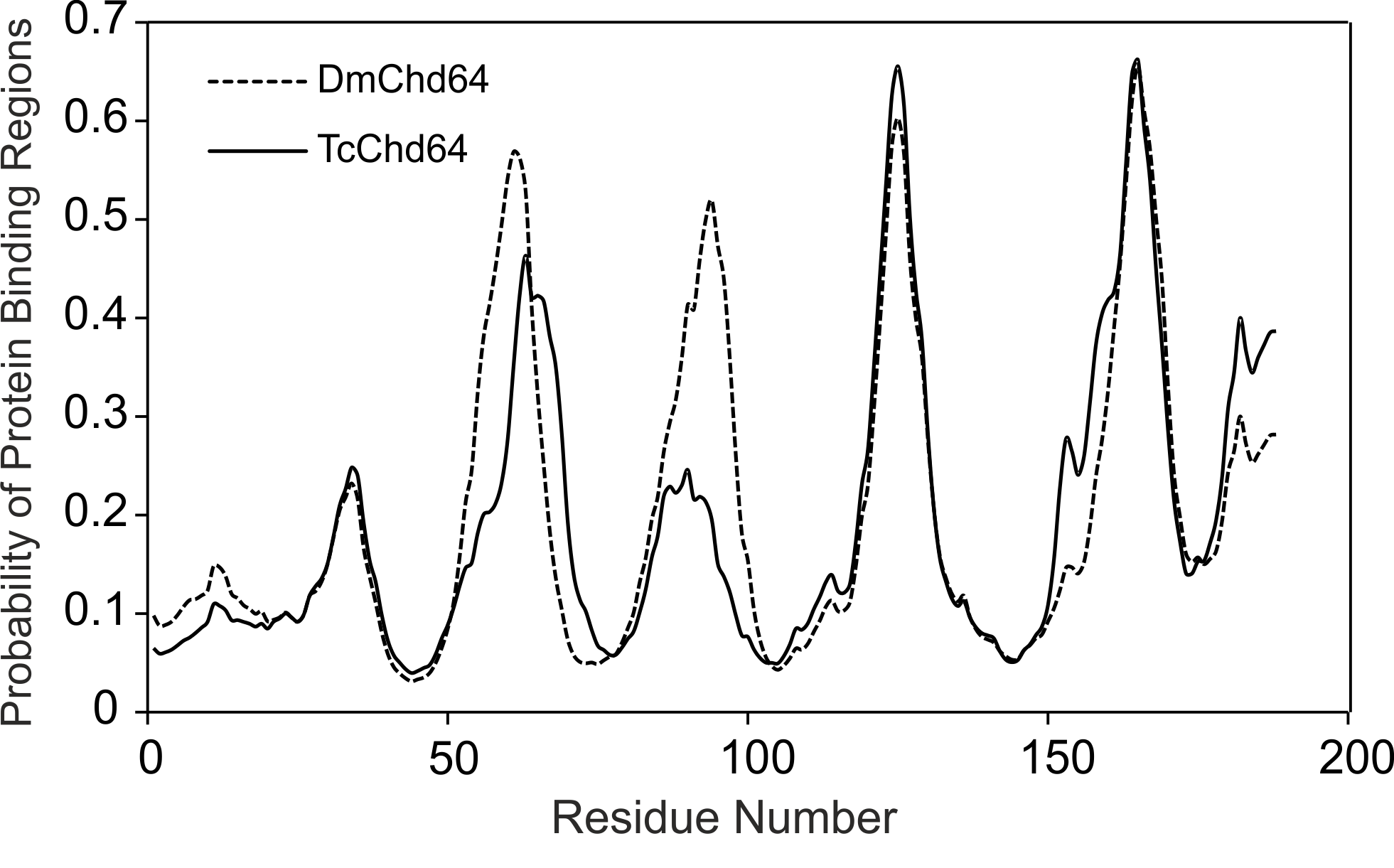

Supplement: Figure S2 — The prediction of protein binding regions in disordered proteins for DmChd64 and TcChd64 by ANCHOR. The prediction of the probability of existence of protein binding regions in DmChd64 (dashed line) and TcChd64 (solid line) was calculated from their primary structure using ANCHOR [46], [47]. The regions with values above 0.5 are potential proteins’ binding regions. (TIF) [file pone.0096809.s002.tif]
